# Sociodemographic factors associated with modern contraceptive use in Japan: an analysis of national survey data

**DOI:** 10.1186/s40834-025-00391-2

**Published:** 2025-10-01

**Authors:** Tasuku Okui

**Affiliations:** Medical Information Center, Kyushu University Hospital, 3-1-1 Maidashi, Higashi-ku, Fukuoka city, Fukuoka prefecture 812-8582 Japan

**Keywords:** Japan, Contraception, Modern contraceptive use, Socioeconomic status, Educational attainment

## Abstract

**Background:**

Few studies have examined the sociodemographic characteristics associated with modern contraceptive use in Japan. This study investigated these associations using data from the National Fertility Survey.

**Methods:**

Data were retrieved from the National Fertility Survey targeting unmarried individuals and married women in Japan. The analysis included unmarried individuals with sexual experience and sexually active married women intending to stop or space childbirths. Modern contraceptive use was examined in relation to sociodemographic characteristics, including age group, number of children, educational attainment, employment status, and income, among unmarried men and women as well as married women. Modified Poisson regression models were employed for the analysis.

**Results:**

Data from 4,874 unmarried individuals and 1,533 married women were used in the analysis. Among both groups, younger age was associated with a higher prevalence of modern contraceptive use. Among unmarried individuals, having one or two children was significantly negatively associated with modern contraceptive use. Conversely, among married women, having multiple children was positively associated with modern contraceptive use. Additionally, a significant negative association was found between being a junior high or high school graduate and modern contraceptive use among unmarried men. Similarly, a significant negative association was observed among married women who were high school graduates. Conversely, modern contraceptive use was significantly positively associated with being an unemployed wife.

**Conclusions:**

Modern contraceptive use in Japan was associated with various sociodemographic characteristics, including the number of children, educational attainment, and employment status.

**Supplementary Information:**

The online version contains supplementary material available at 10.1186/s40834-025-00391-2.

## Background

Contraception plays a vital role in preventing unintended pregnancies and sexually transmitted diseases. Modern contraceptive methods refer to products or medical procedures that prevent pregnancy during sexual intercourse and are typically distinguished from traditional methods, such as fertility awareness and withdrawal. Examples of modern methods include oral contraceptives, condoms, and sterilization [1]. Many studies use modern contraceptive use as an indicator of effective contraceptive or family planning practices [2–8]. The prevalence of modern contraceptive use varies significantly across countries. A study conducted in Latin America and the Caribbean [9] reported that the prevalence of modern contraceptive use among sexually active women of reproductive age was 31.3% and 34.6% in Haiti and Bolivia, respectively, whereas it exceeded 70% in Brazil, Colombia, Costa Rica, Cuba, and Paraguay. According to a study conducted in South Asian countries [10], the prevalence of modern family planning methods among sexually active women of reproductive age was below 50% in the Maldives, Pakistan, and Afghanistan but reached 76% in Bangladesh. Additionally, socioeconomic inequality in access to contraceptives has reportedly improved in low- and middle-income countries in recent years [11, 12]. In the European Union, the prevalence of specific contraceptive methods and access to emergency oral contraceptives vary by country [13, 14]. The use of oral contraceptives exceeded 50% in countries such as Germany and France [14]. In the United States, female sterilization and long-acting reversible contraceptives are used as commonly as oral contraceptives and male condoms [15]. However, approximately one-quarter of women in the United States did not use their preferred contraceptive method [16], with barriers such as logistical issues, lack of knowledge, and cost influencing contraceptive choices. The proportion of individuals with ready access to various contraceptive methods varies across regions worldwide. However, oral contraceptives and condoms tend to be more commonly used than long-acting methods, such as intrauterine devices and sterilization, regardless of region [11]. 

In Japan, 37.5% of married women who intended to stop or space childbirths were not using modern contraceptive methods during sexual intercourse in 2021 [17]. Additionally, among women of reproductive age who used contraception in 2014, the proportions using condoms, the withdrawal method, the rhythm method, and oral contraceptives were 83.4%, 19.5%, 8.3%, and 3.0%, respectively [18]. One notable feature of contraceptive use in Japan is the high prevalence of condom use and the low uptake of other modern contraceptive methods. Condoms are widely available on the market and can be obtained without age restrictions. Conversely, oral contraceptives, intrauterine devices, intrauterine systems, sterilization procedures, and emergency contraceptives require a doctor’s prescription [19, 20]. Moreover, these contraceptive methods are not covered by insurance when used solely for contraception [20], and the cost of oral contraceptives is considered high for young people [21].

Studies worldwide have examined sociodemographic characteristics associated with modern contraceptive use [4–8], identifying variables such as age, number of children, educational attainment, and employment status as significant predictors. However, research on this topic in the Japanese context remains limited. In Japan, a nationally representative survey conducted in 2015 and 2021 showed that modern contraceptive use declined with increasing age among unmarried individuals and married women [17, 22]. Additionally, a web-based questionnaire survey conducted in 2014 among individuals registered with a market research company revealed that lower educational attainment was associated with non-use of condoms or oral contraceptives among both married and unmarried women [23]. However, the number of unmarried women included in that study was only 385, limiting the generalizability of the findings. Moreover, the analysis did not include data on unmarried or married men or explore other socioeconomic factors such as income and employment status. Therefore, investigating the associations between modern contraceptive use and a broader range of sociodemographic characteristics using larger data is essential for formulating effective interventions to promote modern contraceptive use. In this study, we used national survey data to examine the sociodemographic characteristics associated with modern contraceptive use in Japan.

## Material and methods

### Survey data

Data from the National Fertility Survey of unmarried individuals (2015) and married couples (2021) were used in the analysis. These surveys were conducted by the National Institute of Population and Social Security Research, and the institute provided the data in accordance with Article 33 of the Statistics Act in Japan. These surveys targeted unmarried individuals and married women of reproductive age across Japan, respectively, aiming to assess the current status of marriage and childbearing. Although the survey of married couples collected information on both wives and husbands, the responses were provided by the wives. In addition, although a survey of unmarried individuals was also conducted in 2021, it did not include questions on contraceptive use. Similarly, although a survey of married couples conducted in 2015 included questions on contraceptive use, it did not collect information on their sexual activity. Therefore, data from the 2021 survey of unmarried individuals and the 2015 survey of married couples were not used in this study.

The survey of unmarried individuals targeted individuals aged 18–49 years, with 8,752 valid responses obtained from 11,442 distributed questionnaires (response rate: 76.5%) [22]. The survey of married couples targeted married women aged < 55 years, yielding 6,834 valid responses from 9,401 distributed questionnaires (response rate: 72.7%) [17].

### Data processing of the survey data on unmarried individuals

From the survey of unmarried individuals, the study extracted data on birth month and year, sex, educational attainment, employment status, income in the previous year, experience of sexual intercourse with the opposite sex, use of contraception during the most recent sexual encounter, type of contraceptive method used, and number of children born alive.

Only unmarried individuals with sexual intercourse experience responded to the question on contraceptive use. Respondents who reported using contraception selected specific methods: condom, rhythm method/basal body temperature method (hereafter referred to as fertility awareness method), oral contraceptive, withdrawal, or “other” methods. Individuals who used condoms or oral contraceptives were classified as using modern contraceptive methods. Those who reported no contraceptive use or used only fertility awareness or withdrawal methods were classified as not using modern contraceptive methods. Conversely, respondents who used fertility awareness, withdrawal, or other methods in combination with condoms or oral contraceptives were categorized as using modern contraceptive methods. Additionally, because the specific details of “other” methods were unclear, such responses were treated as missing data if modern contraceptive methods were not reported.

Respondents’ ages were calculated based on their birth year and month and categorized into the following groups: < 20 years, 20–24 years, 25–29 years, 30–34 years, 35–39 years, 40–44 years, and 45–49 years. Employment status was classified as regular worker, non-regular worker, self-employed, or unemployed. Educational attainment was categorized as “junior high school,” “high school,” “specialized or professional training college,” “technical college or junior college,” “university or above,” and “others.” Income referred to earnings from employment. As only employed individuals responded to the income question, the income of unemployed individuals was set to zero yen. In the survey, income was reported in 1 million yen increments and was categorized into quantiles for the analysis.

### Data processing of the survey data on married women

The survey targeting married women included data on the wife’s birth year and month, educational attainment and employment status of both spouses, their incomes in the previous year, number of children born to the couple, whether sexual intercourse occurred between the husband and wife in the past month, contraceptive use during the most recent sexual activity, use of modern contraceptives (condom, oral contraceptives, intrauterine device/ring, sterilization, or spermicide), and the couple’s intentions regarding future childbearing. For those intending to have more children, the desired timeframe was also recorded, with response options including “as soon as possible,” “after a while,” “not thinking about it,” and “currently pregnant.” Respondents answered the question on modern contraceptive use with a “yes” or “no”; however, the survey did not collect information on the specific types of modern contraceptive methods used by each couple.

Wives’ ages were categorized into the following groups: < 30 years, 30–34 years, 35–39 years, 40–44 years, and 45–49 years. Because the numbers of wives aged < 20 and 20–24 years were small, those aged < 30 years were combined into a single age group. The couples’ employment statuses and educational levels were categorized in the same manner as for unmarried individuals. Household income was calculated by summing the incomes of the wife and husband, following a previously used method [24], and was categorized into quantiles.

### Inclusion and exclusion criteria

**Fig. 1:**
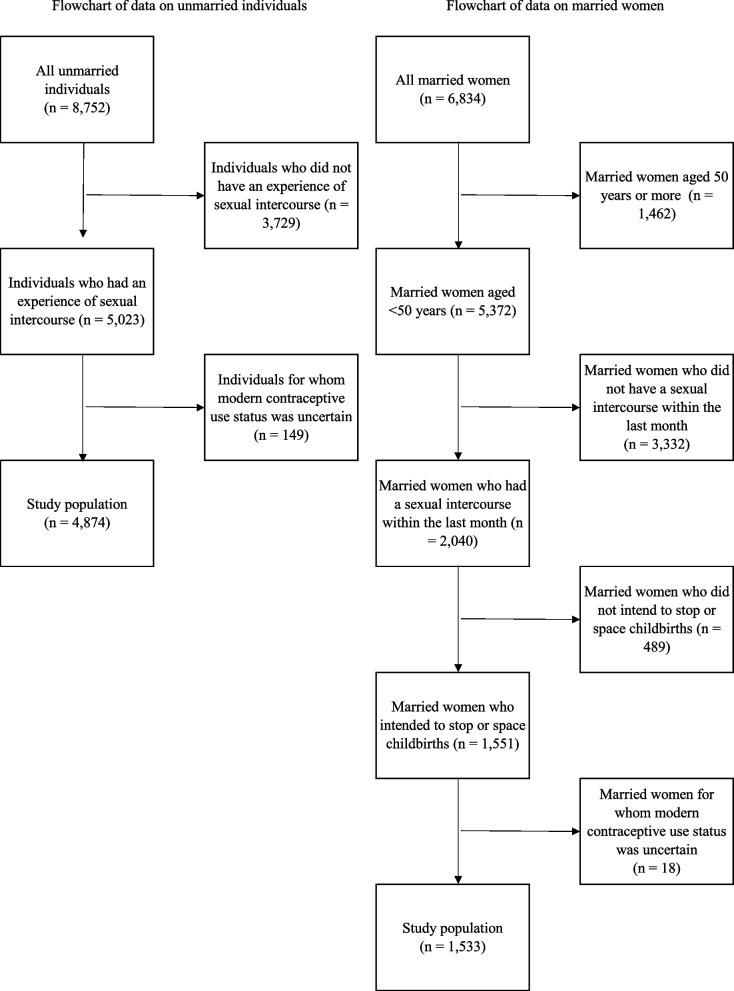
The flowchart of selection of the study population for unmarried individuals and married women

The flowchart for selecting the study populations of unmarried individuals and married women is presented in Fig. 1. For unmarried individuals, only those with experience of sexual intercourse were included in the analysis. Additionally, individuals for whom modern contraceptive use status was uncertain were excluded. Ultimately, a total of 4,874 unmarried individuals were included in the analysis.

Regarding the survey targeting married women, only those who had engaged in sexual intercourse within the previous month were included in the analysis. Additionally, the study was limited to married women aged < 50 years. To focus on individuals with a need for contraception, the analysis further included only those who intended to stop or space childbirths [25]. This group comprised women who reported no intention to have additional children as well as those who intended to have more children after a while. This definition is consistent with that used by the National Institute of Population and Social Security Research [17]. Additionally, married women for whom the status of modern contraceptive use was uncertain were excluded from the analysis. Consequently, a total of 1,533 married women were included in the study.

### Statistical analysis

Analyses were conducted separately for unmarried individuals and married women. For unmarried individuals, analyses were conducted by sex. The number and proportion of individuals using modern contraceptive methods were calculated according to sociodemographic characteristics for both groups. Additionally, a modified Poisson regression model was employed to examine the association between modern contraceptive use and sociodemographic characteristics. In the analysis of unmarried individuals, the explanatory variables included age group, number of children, educational attainment, employment status, and income. For married women, the explanatory variables included the wife’s age group, number of children, household income, and the couple’s educational attainments and employment statuses.

Prevalence ratios (PRs), robust standard errors, 95% confidence intervals (CIs), and *p*-values were calculated for each explanatory variable, with a *p*-value of < 0.05 considered statistically significant.

To address missing data, the study conducted both a complete-case analysis and a multiple imputation analysis as a sensitivity analysis. In the complete-case analysis, respondents with missing values were excluded, whereas in the multiple imputation analysis, missing data were imputed. Multiple imputation was performed using chained equations with predictive mean matching [26], and 50 imputations were generated. All analyses were conducted using R version 4.1.3 [27], with the car, lmtest, mice, and sandwich packages [26, 28–30]. The statistics presented in this study were not published by the National Institute; rather, they were independently generated through analysis of the survey data provided by the institute.

## Results

### Proportion of modern contraceptive use among unmarried individuals according to sociodemographic characteristics

Table 1 presents the number and proportion of unmarried men using modern contraceptives according to sociodemographic characteristics. The proportion of modern contraceptive use tended to decrease with increasing age. It was lower among those with children than among those without. Additionally, modern contraceptive use tended to increase with higher educational attainments.

**Table 1:** Number and proportion of unmarried men with modern contraceptive use by sex and sociodemographic characteristics

	Men			
Characteristics	Number (%) of all men ^a^	Number (%) of men without modern contraceptive use ^a^	Number (%) of men with modern contraceptive use ^a^	Proportion of men with modern contraceptive use (%) ^b^
Total	2,563 (100.0)	480 (100.0)	2,083 (100.0)	81.3
Age group				
Under 20 years	97 (3.8)	12 (2.5)	85 (4.1)	87.6
20–24 years	443 (17.3)	46 (9.6)	397 (19.1)	89.6
25–29 years	509 (19.9)	54 (11.2)	455 (21.8)	89.4
30–34 years	423 (16.5)	74 (15.4)	349 (16.8)	82.5
35–39 years	361 (14.1)	100 (20.8)	261 (12.5)	72.3
40–44 years	393 (15.3)	96 (20.0)	297 (14.3)	75.6
45–49 years	337 (13.1)	98 (20.4)	239 (11.5)	70.9
Number of children				
0	2,199 (85.8)	355 (74.0)	1,844 (88.5)	83.9
1	83 (3.2)	35 (7.3)	48 (2.3)	57.8
2	83 (3.2)	42 (8.8)	41 (2.0)	49.4
3 or more	29 (1.1)	9 (1.9)	20 (1.0)	69.0
Missing	169 (6.6)	39 (8.1)	130 (6.2)	76.9
Educational attainment				
Junior high school	135 (5.3)	47 (9.8)	88 (4.2)	65.2
High school	905 (35.3)	240 (50.0)	665 (31.9)	73.5
Specialized or professional training college	363 (14.2)	65 (13.5)	298 (14.3)	82.1
Technical college or junior college	65 (2.5)	9 (1.9)	56 (2.7)	86.2
University or more	838 (32.7)	95 (19.8)	743 (35.7)	88.7
Others	211 (8.2)	13 (2.7)	198 (9.5)	93.8
Missing	46 (1.8)	11 (2.3)	35 (1.7)	76.1
Employment status				
Regular worker	1,476 (57.6)	284 (59.2)	1,192 (57.2)	80.8
Non–regular worker	359 (14.0)	67 (14.0)	292 (14.0)	81.3
Self–employed worker	196 (7.6)	48 (10.0)	148 (7.1)	75.5
Unemployed person	427 (16.7)	63 (13.1)	364 (17.5)	85.2
Missing	105 (4.1)	18 (3.8)	87 (4.2)	82.9
Income				
Quantile 1(Lowest)	591 (23.1)	89 (18.5)	502 (24.1)	84.9
Quantile 2	628 (24.5)	133 (27.7)	495 (23.8)	78.8
Quantile 3	511 (19.9)	108 (22.5)	403 (19.3)	78.9
Quantile 4 (Highest)	647 (25.2)	121 (25.2)	526 (25.3)	81.3
Missing	186 (7.3)	29 (6.0)	157 (7.5)	84.4
Contraceptive method ^c^				
Condom	2,057 (80.3)	0 (0.0)	2,057 (98.8)	100.0
Oral contraceptive	45 (1.8)	0 (0.0)	45 (2.2)	100.0
Fertility awareness	10 (0.4)	3 (0.6)	7 (0.3)	70.0
Withdrawal	121 (4.7)	80 (16.7)	41 (2.0)	33.9
Others	2 (0.1)	0 (0.0)	2 (0.1)	100.0
No contraception	397 (15.5)	397 (82.7)	0 (0.0)	0.0

^a^ The proportion (%) indicates column percentage

^b^ The proportion (%) indicates row percentage

^c^ Respondents could select multiple contraceptive methods. Some respondents used the fertility awareness methods, the withdrawal methods, and the other methods in addition to the modern contraceptive methods (condoms and oral contraceptives), and they were categorized as persons with modern contraceptive use

Table 2 shows the number and proportion of unmarried women using modern contraceptives by sociodemographic characteristics. The results showed similar trends to those observed among men.

**Table 2:** Number and proportion of unmarried women with modern contraceptive use by sex and sociodemographic characteristics

	Women			
Characteristics	Number (%) of all women ^a^	Number (%) of women without modern contraceptive use ^a^	Number (%) of women with modern contraceptive use ^a^	Proportion of women with modern contraceptive use (%) ^b^
Total	2,311 (100.0)	594 (100.0)	1,717 (100.0)	74.3
Age group				
Under 20 years	77 (3.3)	11 (1.9)	66 (3.8)	85.7
20–24 years	508 (22.0)	71 (12.0)	437 (25.5)	86.0
25–29 years	480 (20.8)	85 (14.3)	395 (23.0)	82.3
30–34 years	316 (13.7)	91 (15.3)	225 (13.1)	71.2
35–39 years	277 (12.0)	91 (15.3)	186 (10.8)	67.1
40–44 years	346 (15.0)	109 (18.4)	237 (13.8)	68.5
45–49 years	307 (13.3)	136 (22.9)	171 (10.0)	55.7
Number of children				
0	1,730 (74.9)	371 (62.5)	1,359 (79.1)	78.6
1	225 (9.7)	95 (16.0)	130 (7.6)	57.8
2	198 (8.6)	83 (14.0)	115 (6.7)	58.1
3 or more	78 (3.4)	31 (5.2)	47 (2.7)	60.3
Missing	80 (3.5)	14 (2.4)	66 (3.8)	82.5
Educational attainment				
Junior high school	107 (4.6)	45 (7.6)	62 (3.6)	57.9
High school	713 (30.9)	217 (36.5)	496 (28.9)	69.6
Specialized or professional training college	437 (18.9)	119 (20.0)	318 (18.5)	72.8
Technical college or junior college	314 (13.6)	87 (14.6)	227 (13.2)	72.3
University or more	548 (23.7)	99 (16.7)	449 (26.2)	81.9
Others	170 (7.4)	19 (3.2)	151 (8.8)	88.8
Missing	22 (1.0)	8 (1.3)	14 (0.8)	63.6
Employment status				
Regular worker	1,168 (50.5)	259 (43.6)	909 (52.9)	77.8
Non–regular worker	654 (28.3)	203 (34.2)	451 (26.3)	69.0
Self–employed worker	68 (2.9)	24 (4.0)	44 (2.6)	64.7
Unemployed person	348 (15.1)	83 (14.0)	265 (15.4)	76.1
Missing	73 (3.2)	25 (4.2)	48 (2.8)	65.8
Income				
Quantile 1(Lowest)	566 (24.5)	138 (23.2)	428 (24.9)	75.6
Quantile 2	1,037 (44.9)	269 (45.3)	768 (44.7)	74.1
Quantile 3	344 (14.9)	82 (13.8)	262 (15.3)	76.2
Quantile 4 (Highest)	220 (9.5)	60 (10.1)	160 (9.3)	72.7
Missing	144 (6.2)	45 (7.6)	99 (5.8)	68.8
Contraceptive method ^c^				
Condom	1,658 (71.7)	0 (0.0)	1,658 (96.6)	100.0
Oral contraceptive	111 (4.8)	0 (0.0)	111 (6.5)	100.0
Fertility awareness	22 (1.0)	8 (1.3)	14 (0.8)	63.6
Withdrawal	192 (8.3)	139 (23.4)	53 (3.1)	27.6
Others	1 (0.0)	0 (0.0)	1 (0.1)	100.0
No contraception	449 (19.4)	449 (75.6)	0 (0.0)	0.0

^a^ The proportion (%) indicates column percentage

^b^ The proportion (%) indicates row percentage

^c^ Respondents could select multiple contraceptive methods. Some respondents used the fertility awareness methods, the withdrawal methods, and the other methods in addition to the modern contraceptive methods (condoms and oral contraceptives), and they were categorized as persons with modern contraceptive use

### Proportion of modern contraceptive use among married women according to sociodemographic characteristics

Table 3 presents the number and proportion of married women using modern contraceptives according to sociodemographic characteristics. The proportion of modern contraceptive use tended to decline with increasing age of the wife and was higher among women with two or more children than in those with one or no children. Modern contraceptive use also tended to increase with higher educational attainment of both wives and husbands. Regarding employment status, the highest proportion of modern contraceptive use was observed among unemployed wives and among husbands who were regular workers.

**Table 3:** Number and proportion of married women with modern contraceptive use by sociodemographic characteristics

Characteristics	Number (%) of all women ^a^	Number (%) of women without modern contraceptive use ^a^	Number (%) of women with modern contraceptive use ^a^	Proportion of women with modern contraceptive use (%) ^b^
Total	1533 (100.0)	639 (100.0)	894 (100.0)	58.3
Age group of wife				
Under 30 years	138 (9.0)	37 (5.8)	101 (11.3)	73.2
30–34 years	204 (13.3)	76 (11.9)	128 (14.3)	62.7
35–39 years	378 (24.7)	163 (25.5)	215 (24.0)	56.9
40–44 years	434 (28.3)	179 (28.0)	255 (28.5)	58.8
45–49 years	379 (24.7)	184 (28.8)	195 (21.8)	51.5
Number of children				
0	128 (8.3)	66 (10.3)	62 (6.9)	48.4
1	232 (15.1)	118 (18.5)	114 (12.8)	49.1
2	729 (47.6)	276 (43.2)	453 (50.7)	62.1
3 or more	426 (27.8)	169 (26.4)	257 (28.7)	60.3
Missing	18 (1.2)	10 (1.6)	8 (0.9)	44.4
Educational attainment of wife				
Junior high school	40 (2.6)	20 (3.1)	20 (2.2)	50.0
High school	459 (29.9)	216 (33.8)	243 (27.2)	52.9
Specialized or professional training college	328 (21.4)	136 (21.3)	192 (21.5)	58.5
Technical college or junior college	271 (17.7)	110 (17.2)	161 (18.0)	59.4
University or more	389 (25.4)	140 (21.9)	249 (27.9)	64.0
Others	10 (0.7)	3 (0.5)	7 (0.8)	70.0
Missing	36 (2.3)	14 (2.2)	22 (2.5)	61.1
Educational attainment of husband				
Junior high school	77 (5.0)	43 (6.7)	34 (3.8)	44.2
High school	530 (34.6)	239 (37.4)	291 (32.6)	54.9
Specialized or professional training college	225 (14.7)	88 (13.8)	137 (15.3)	60.9
Technical college or junior college	41 (2.7)	16 (2.5)	25 (2.8)	61.0
University or more	610 (39.8)	231 (36.2)	379 (42.4)	62.1
Others	4 (0.3)	3 (0.5)	1 (0.1)	25.0
Missing	46 (3.0)	19 (3.0)	27 (3.0)	58.7
Employment status of wife				
Regular worker	469 (30.6)	209 (32.7)	260 (29.1)	55.4
Non–regular worker	597 (38.9)	247 (38.7)	350 (39.1)	58.6
Self–employed worker	93 (6.1)	44 (6.9)	49 (5.5)	52.7
Unemployed person	333 (21.7)	123 (19.2)	210 (23.5)	63.1
Missing	41 (2.7)	16 (2.5)	25 (2.8)	61.0
Employment status of husband				
Regular worker	1254 (81.8)	510 (79.8)	744 (83.2)	59.3
Non–regular worker	38 (2.5)	22 (3.4)	16 (1.8)	42.1
Self–employed worker	175 (11.4)	77 (12.1)	98 (11.0)	56.0
Unemployed person	15 (1.0)	8 (1.3)	7 (0.8)	46.7
Missing	51 (3.3)	22 (3.4)	29 (3.2)	56.9
Household income				
Quantile 1(Lowest)	391 (25.5)	169 (26.4)	222 (24.8)	56.8
Quantile 2	400 (26.1)	161 (25.2)	239 (26.7)	59.8
Quantile 3	311 (20.3)	119 (18.6)	192 (21.5)	61.7
Quantile 4 (Highest)	284 (18.5)	129 (20.2)	155 (17.3)	54.6
Missing	147 (9.6)	61 (9.5)	86 (9.6)	58.5
Contraception				
Yes	993 (64.8)	99 (15.5)	894 (100.0)	90.0
No	540 (35.2)	540 (84.5)	0 (0.0)	0.0

^a^ The proportion (%) indicates column percentage

^b^ The proportion (%) indicates row percentage

### Association between sociodemographic characteristics and modern contraceptive use among unmarried individuals

Table 4 presents the results of the regression analysis examining associations between sociodemographic characteristics and modern contraceptive use among unmarried individuals. Statistically significant positive associations were observed between younger age groups and modern contraceptive use for both men and women. Additionally, having one or two children was significantly negatively associated with modern contraceptive use. Among men, the adjusted PRs were 0.69 (95% CI: 0.55–0.86) for those with one child and 0.63 (95% CI: 0.50–0.81) for those with two children. Among women, the corresponding adjusted PRs were 0.82 (95% CI: 0.72–0.93) and 0.87 (95% CI: 0.76–0.99), respectively. The study also revealed statistically significant negative associations between being a junior high school or high school graduate and modern contraceptive use, with adjusted PRs of 0.75 (95% CI:0.64–0.87) and 0.87 (95% CI:0.83–0.91), respectively. Furthermore, a statistically significant negative association was observed between the second income quantile and modern contraceptive use among men, with an adjusted PR of 0.93 (95% CI:0.87–0.99).

**Table 4:** Results of the regression analysis investigating associations between sociodemographic characteristics and modern contraceptive use for unmarried persons

	Men		Women	
Characteristics	Adjusted PR (95%CI) ^a^	*p*–value	Adjusted PR (95%CI) ^a^	*p*–value
Age group				
Under 20 years	1.11 (0.98, 1.26)	0.099	1.39 (1.19, 1.62)	** < 0.001**
20–24 years	1.17 (1.07, 1.28)	** < 0.001**	1.39 (1.23, 1.57)	** < 0.001**
25–29 years	1.16 (1.06, 1.26)	** < 0.001**	1.33 (1.18, 1.51)	** < 0.001**
30–34 years	1.10 (1.01, 1.20)	**0.031**	1.22 (1.07, 1.39)	**0.003**
35–39 years	0.98 (0.89, 1.08)	0.636	1.18 (1.03, 1.35)	**0.018**
40–44 years	1.04 (0.94, 1.14)	0.479	1.21 (1.06, 1.38)	**0.004**
45–49 years	Reference		Reference	
Number of children				
0	Reference		Reference	
1	0.69 (0.55, 0.86)	** < 0.001**	0.82 (0.72, 0.93)	**0.002**
2	0.63 (0.50, 0.81)	** < 0.001**	0.87 (0.76, 0.99)	**0.041**
3 or more	0.95 (0.73, 1.23)	0.676	0.91 (0.75, 1.11)	0.368
Educational attainment				
Junior high school	0.75 (0.64, 0.87)	** < 0.001**	0.84 (0.69, 1.01)	0.063
High school	0.87 (0.83, 0.91)	** < 0.001**	0.97 (0.90, 1.04)	0.344
Specialized or professional training college	0.95 (0.90, 1.01)	0.108	0.95 (0.88, 1.02)	0.167
Technical college or junior college	1.00 (0.90, 1.12)	0.963	0.94 (0.87, 1.02)	0.163
University or more	Reference		Reference	
Others	0.97 (0.89, 1.05)	0.448	1.10 (0.97, 1.25)	0.145
Employment status				
Regular worker	Reference		Reference	
Non–regular worker	1.05 (0.98, 1.12)	0.137	0.96 (0.89, 1.02)	0.177
Self–employed worker	1.00 (0.92, 1.10)	0.945	0.98 (0.82, 1.17)	0.791
Unemployed person	1.06 (0.96, 1.17)	0.253	0.91 (0.80, 1.04)	0.181
Income				
Quantile 1(Lowest)	0.95 (0.87, 1.03)	0.212	1.00 (0.88, 1.13)	0.997
Quantile 2	0.93 (0.87, 0.99)	**0.027**	1.01 (0.92, 1.11)	0.810
Quantile 3	0.96 (0.90, 1.02)	0.175	0.99 (0.90, 1.10)	0.912
Quantile 4 (Highest)	Reference		Reference	

*PR* prevalence ratio, *CI* confidence interval

^a^ PR represents the prevalence ratio of modern contraceptive use for each category relative to the reference category

Supplementary Table 1 presents the results of the regression analysis examining the associations between sociodemographic characteristics and modern contraceptive use among unmarried individuals using multiple imputation. The findings were generally consistent with those from the complete-case analysis; however, a statistically significant negative association was observed between being a junior high school graduate and modern contraceptive use among women.

### Association between sociodemographic characteristics and modern contraceptive use among married women

Table 5 presents the results of the regression analysis examining the associations between sociodemographic characteristics and modern contraceptive use among married women. A statistically significant positive association was found between the age group “less than 30 years” and modern contraceptive use, with an adjusted PR of 1.77 (95% CI:1.49–2.10). Additionally, statistically significant positive associations were observed between having two children and three or more children and modern contraceptive use, with adjusted PRs of 1.32 (95% CI:1.08–1.61) and 1.34 (95% CI:1.08–1.66), respectively. Moreover, the results showed a statistically significant negative association between wives who were high school graduates and modern contraceptive use, with an adjusted PR of 0.84 (95% CI:0.73–0.97). In contrast, statistically significant positive associations were observed between modern contraceptive use and wives who were non-regular workers or unemployed, with adjusted PRs of 1.18 (95% CI:1.05–1.34) and 1.22 (95% CI:1.07–1.40), respectively.

**Table 5:** Results of the regression analysis investigating associations between sociodemographic characteristics and modern contraceptive use for married women

Characteristics	Adjusted PR (95%CI) ^a^	*p*–value
Age group of wife		
Under 30 years	1.77 (1.49, 2.10)	** < 0.001**
30–34 years	1.18 (1.00, 1.39)	0.056
35–39 years	1.08 (0.93, 1.25)	0.321
40–44 years	1.09 (0.95, 1.25)	0.235
45–49 years	Reference	
Number of children		
0	Reference	
1	0.94 (0.75, 1.18)	0.588
2	1.32 (1.08, 1.61)	**0.007**
3 or more	1.34 (1.08, 1.66)	**0.008**
Educational attainment of wife		
Junior high school	0.97 (0.69, 1.35)	0.851
High school	0.84 (0.73, 0.97)	**0.020**
Specialized or professional training college	0.93 (0.81, 1.06)	0.256
Technical college or junior college	0.91 (0.79, 1.05)	0.195
University or more	Reference	
Others	0.86 (0.49, 1.51)	0.608
Educational attainment of husband		
Junior high school	0.79 (0.59, 1.06)	0.115
High school	0.91 (0.81, 1.03)	0.144
Specialized or professional training college	1.02 (0.88, 1.17)	0.827
Technical college or junior college	1.00 (0.75, 1.33)	0.993
University or more	Reference	
Others	0.41 (0.04, 3.76)	0.431
Employment status of wife		
Regular worker	Reference	
Non–regular worker	1.18 (1.04, 1.34)	**0.008**
Self–employed worker	0.99 (0.76, 1.29)	0.951
Unemployed person	1.22 (1.07, 1.40)	**0.004**
Employment status of husband		
Regular worker	Reference	
Non–regular worker	0.70 (0.46, 1.07)	0.096
Self–employed worker	1.03 (0.87, 1.23)	0.713
Unemployed person	0.95 (0.49, 1.82)	0.872
Household income		
Quantile 1(Lowest)	0.99 (0.84, 1.17)	0.937
Quantile 2	1.00 (0.86, 1.17)	0.969
Quantile 3	1.08 (0.94, 1.25)	0.278
Quantile 4 (Highest)	Reference	

*PR* prevalence ratio, *CI* confidence interval

^a^ PR represents the prevalence ratio of modern contraceptive use for each category relative to the reference category

Supplementary Table 2 presents the results of the regression analysis examining the associations between sociodemographic characteristics and modern contraceptive use among married women using multiple imputation. The findings were generally consistent with those from the complete-case analysis; however, the association between being a non-regular worker and modern contraceptive use among wives was not statistically significant.

## Discussion

This study examined the associations between sociodemographic characteristics and modern contraceptive use among unmarried individuals and married women in Japan. Both complete-case and multiple imputation analyses were conducted to address missing data. As the results showed slight differences between the two approaches, the discussion primarily focuses on statistically significant associations that were consistent across both methods, highlighting the most robust findings.

The study found a higher prevalence of modern contraceptive use among unmarried individuals and married women in younger age groups. Similar findings have been reported in studies conducted in Liberia, Canada, and Spain [6, 25, 31], and perimenopausal symptoms and reduced fecundity among older women have been suggested as possible explanations for this trend [6]. Conversely, studies in the United States have reported lower prevalence of contraceptive use among younger women of reproductive age [15, 32]. In Japan, withdrawal remains a common method among older adults [22], suggesting the presence of a birth cohort effect in contraceptive method preferences.

The study found a negative association between having one or two children and modern contraceptive use among unmarried individuals, whereas a positive association was observed between having multiple children and modern contraceptive use among married women. This finding is consistent with results from studies conducted in Liberia, Brazil, the United States, Denmark, Germany, Poland, Italy, and Spain, wherein nulliparous women were found to have lower prevalence of (modern) contraceptive use [6, 33–35]. Additionally, a study across 73 low- and middle-income countries reported that the prevalence of modern contraceptive use was lowest among married women without children when compared with unmarried women and married women with children [36], which is consistent with the findings of this study. It is suggested that the need for contraception increases among married women who feel that they cannot afford to have additional children [37]. Conversely, the negative association between the number of children and modern contraceptive use among unmarried individuals may reflect a different dynamic. One possible hypothesis is that unmarried individuals who did not use modern contraceptives were more likely to have children, which may have contributed to the observed association.

The study also identified a negative association between lower educational attainment and modern contraceptive use among unmarried men. Although the association was not statistically significant among unmarried women, the estimated PR for junior high school graduates suggested a similar trend. Among married women, a negative association was observed for those who were high school graduates, whereas no significant association was found for those with only junior high school education, likely due to the small number of participants in this category. A previous study in Japan reported that both married and unmarried women with lower educational attainment had lower prevalence of modern contraceptive use [23]. The present study extends these findings by showing that the association persists among married women even after adjusting for the characteristics of their husbands and other socioeconomic factors. Several studies conducted in Uganda, Yemen, the United States, Canada, Denmark, and Spain [7, 25, 31, 34, 35, 38] have observed similar associations among women, attributing the relationship to the impact of education on health-seeking behaviors [38]. An association between health-seeking behavior and educational attainment has also been observed in Japan [39]. Additionally, a study in Ghana found that higher educational attainment among men was associated with modern contraceptive use [3], although research on the relationship between men’s educational attainment and contraceptive use remains limited. A notable characteristic of contraceptive use in Japan is the overwhelmingly high prevalence of condom use. Furthermore, female (internal) condoms have been discontinued in Japan since 2011 [40], leaving only male (external) condoms available. Consequently, men’s educational attainment may have a greater influence on modern contraceptive use in Japan than in other countries.

Unemployed wives exhibited a higher prevalence of modern contraceptive use than those who were regular workers. Previous studies conducted in Liberia, Tanzania, Bangladesh, Ethiopia, Germany, and Canada have indicated that working women tend to use modern contraceptives more than unemployed women [6, 25, 34, 41–43], whereas in Denmark, unemployed women were more likely to use contraceptives [34]. One explanation for this finding is that employed women tend to have higher educational attainments, better income, and improved access to healthcare [6, 43]. Although the underlying reason for the association observed in our study remains unclear, a previous study indicated that working women may be more likely to forget to take oral contraceptives [44]. However, given the low prevalence of oral contraceptive use in Japan [18], other factors are likely influencing this result. One hypothesis is that unemployed wives have more available time, which may facilitate greater access to modern contraceptives. Another possibility is that some unemployed wives may refrain from having more children due to illness, childrearing, or caregiving, which could contribute to the higher prevalence of modern contraceptive use. Additionally, a negative association was found between being in the second income quantile and modern contraceptive use among unmarried men, whereas the PRs for income quantiles 1–4 were similar among women. Although this significant association was observed only in the second quantile for men, the PR estimates for quantiles 1–3 were all below one and relatively similar. The reason for this association appearing solely among men remains unknown. Previous studies conducted in Ghana, Canada, and Pakistan have similarly reported negative associations between lower household income and (modern) contraceptive use [25, 45, 46].

Among husbands, lower educational attainment has been linked to higher frequency of unintended pregnancies in Japan [24], which may be related to the lower modern contraceptive use observed among unmarried men with lower educational attainment. To reduce such inequalities, contraception education may need to be introduced earlier in the educational process. Additionally, workplace and community-based educational programs could help reach individuals with lower educational attainment, who may have fewer opportunities to learn about modern contraceptive methods. Improving access to modern contraceptives, such as intrauterine devices and oral contraceptives, through expanded insurance coverage may also help address disparities. Furthermore, the finding that unemployed wives have a higher prevalence of modern contraceptive use than regular workers warrants further investigation to understand the underlying reasons.

This study has some measurement- and design-related limitations. A measurement-related limitation is that for unmarried individuals, only condom and oral contraceptive use were included as modern contraceptive methods in the analysis. This was because the survey questionnaire specifically asked about condom use, fertility awareness methods, oral contraceptives, and withdrawal methods. However, data from 2015 indicate that the use of other modern contraceptive methods among unmarried individuals aged 18–34 years was very low, 0.1% for men and 0.2% for women in Japan [22], suggesting that few unmarried individuals used modern contraceptive methods other than condoms and oral contraceptives. Additionally, differences existed in the survey questions regarding contraceptive use and sexual experience between unmarried individuals and married women. Conducting surveys with standardized questions across these groups would improve comparability. Furthermore, as the National Fertility Survey relies on self-reported data, there is potential for over-reporting of modern contraceptive use due to social desirability bias [47], which can occur even in anonymous surveys [48]. This bias may vary according to sociodemographic factors such as age and marital status [49], potentially influencing the study’s results. Regarding design limitations, this study used data from unmarried individuals surveyed in 2015 and married women surveyed in 2021. The difference in survey years presents a limitation, as older birth cohorts tend to have lower modern contraceptive use, and the prevalence of modern contraceptive use among unmarried individuals in 2015 may have been lower than it would be in 2021.

## Conclusions

This study demonstrated that modern contraceptive use in Japan was associated with sociodemographic factors such as number of children, educational attainment, and employment status. Notably, unlike findings from previous studies in other countries, a positive association was observed between being an unemployed wife and modern contraceptive use. Consistent with international evidence, lower educational attainment among wives was associated with lower prevalence of modern contraceptive use. However, this study also identified a similar association among unmarried men, a finding that has not been widely explored or reported in other countries. These results suggest the need for targeted educational programs for individuals with lower education level and broader insurance coverage for modern contraceptive methods to reduce the disparities. Additionally, future research should explore the underlying mechanisms driving these associations. 

## Supplementary Information

Supplementary Material 1. Table 1. Results of the regression analysis investigating associations between sociodemographic characteristics and modern contraceptive use for unmarried individuals using multiple imputation. Table 2. Results of the regression analysis investigating associations between sociodemographic characteristics and modern contraceptive use for married women using multiple imputation.

## Data Availability

The data that support the findings of this study are available from the National Institute of Population and Social Security Research in Japan. Restrictions apply to the availability of these data, which were used under license for this study and are not publicly available. Data are available from the National Institute of Population and Social Security Research if the Institute permits use of the data.
