# Supplementary material for: Sociodemographic factors associated with modern contraceptive use in Japan: an analysis of national survey data
Source: Contracept Reprod Med. 2025 Oct 1;10:65. doi: 10.1186/s40834-025-00391-2 (PMC12486691; doi:10.1186/s40834-025-00391-2)
Supplement: Supplementary file 1 — Supplementary Material 1. Table 1. Results of the regression analysis investigating associations between sociodemographic characteristics and modern contraceptive use for unmarried individuals using multiple imputation. Table 2. Results of the regression analysis investigating associations between sociodemographic characteristics and modern contraceptive use for married women using multiple imputation. [file 40834_2025_391_MOESM1_ESM.docx]

Supplementary table 1. Results of the regression analysis investigating associations between sociodemographic characteristics and modern contraceptive use for unmarried individuals using multiple imputation.

|  | Men | | Women | |
| --- | --- | --- | --- | --- |
| Characteristics | Adjusted PR (95%CI) ^a^ | p–value | Adjusted PR (95%CI) ^a^ | p–value |
| Age group |  |  |  |  |
| Under 20 years | 1.14 (1.02, 1.28) | **0.019** | 1.38 (1.18, 1.61) | **<0.001** |
| 20–24 years | 1.17 (1.08, 1.28) | **<0.001** | 1.39 (1.23, 1.56) | **<0.001** |
| 25–29 years | 1.16 (1.07, 1.26) | **<0.001** | 1.35 (1.20, 1.51) | **<0.001** |
| 30–34 years | 1.09 (1.01, 1.19) | **0.032** | 1.21 (1.07, 1.37) | **0.003** |
| 35–39 years | 0.98 (0.89, 1.07) | 0.600 | 1.16 (1.02, 1.32) | **0.026** |
| 40–44 years | 1.05 (0.96, 1.15) | 0.245 | 1.21 (1.07, 1.38) | **0.002** |
| 45–49 years | Reference |  | Reference |  |
| Number of children |  |  |  |  |
| 0 | Reference |  | Reference |  |
| 1 | 0.78 (0.65, 0.93) | **0.006** | 0.82 (0.73, 0.93) | **0.001** |
| 2 | 0.67 (0.54, 0.84) | **<0.001** | 0.86 (0.76, 0.98) | **0.029** |
| 3 or more | 0.92 (0.70, 1.20) | 0.517 | 0.92 (0.76, 1.12) | 0.403 |
| Educational attainment |  |  |  |  |
| Junior high school | 0.79 (0.70, 0.90) | **<0.001** | 0.84 (0.70, 1.00) | **0.045** |
| High school | 0.87 (0.83, 0.91) | **<0.001** | 0.95 (0.89, 1.02) | 0.186 |
| Specialized or professional training college | 0.96 (0.91, 1.01) | 0.119 | 0.95 (0.88, 1.02) | 0.147 |
| Technical college or junior college | 1.00 (0.90, 1.11) | 0.965 | 0.95 (0.88, 1.03) | 0.183 |
| University or more | Reference |  | Reference |  |
| Others | 0.97 (0.89, 1.06) | 0.508 | 1.10 (0.97, 1.25) | 0.132 |
| Employment status |  |  |  |  |
| Regular worker | Reference |  | Reference |  |
| Non–regular worker | 1.05 (0.98, 1.11) | 0.141 | 0.95 (0.89, 1.02) | 0.141 |
| Self–employed worker | 1.00 (0.92, 1.09) | 0.965 | 0.93 (0.78, 1.12) | 0.446 |
| Unemployed person | 1.04 (0.94, 1.15) | 0.454 | 0.90 (0.79, 1.03) | 0.134 |
| Income |  |  |  |  |
| Quantile 1(Lowest) | 0.96 (0.88, 1.04) | 0.312 | 1.01 (0.89, 1.14) | 0.899 |
| Quantile 2 | 0.94 (0.88, 1.00) | **0.047** | 1.01 (0.92, 1.11) | 0.839 |
| Quantile 3 | 0.95 (0.90, 1.01) | 0.098 | 1.00 (0.90, 1.11) | 0.982 |
| Quantile 4 (Highest) | Reference |  | Reference |  |
| PR, prevalence ratio; CI, confidence interval |  |  |  |  |
| ^a^ PR represents the prevalence ratio of modern contraceptive use for each category relative to the reference category. | | | | |

Supplementary table 2. Results of the regression analysis investigating associations between sociodemographic characteristics and modern contraceptive use for married women using multiple imputation.

| Characteristics | Adjusted PR (95%CI) ^a^ | p–value |
| --- | --- | --- |
| Age group of wife |  |  |
| Under 30 years | 1.65 (1.40, 1.94) | **<0.001** |
| 30–34 years | 1.19 (1.02, 1.38) | **0.026** |
| 35–39 years | 1.06 (0.92, 1.21) | 0.409 |
| 40–44 years | 1.09 (0.96, 1.24) | 0.164 |
| 45–49 years | Reference |  |
| Number of children |  |  |
| 0 | Reference |  |
| 1 | 1.01 (0.81, 1.25) | 0.934 |
| 2 | 1.41 (1.16, 1.71) | **<0.001** |
| 3 or more | 1.40 (1.15, 1.72) | **0.001** |
| Educational attainment of wife |  |  |
| Junior high school | 0.80 (0.57, 1.13) | 0.207 |
| High school | 0.84 (0.74, 0.96) | **0.010** |
| Specialized or professional training college | 0.89 (0.79, 1.01) | 0.074 |
| Technical college or junior college | 0.92 (0.80, 1.05) | 0.198 |
| University or more | Reference |  |
| Others | 0.93 (0.61, 1.42) | 0.731 |
| Educational attainment of husband |  |  |
| Junior high school | 0.77 (0.59, 1.01) | 0.055 |
| High school | 0.92 (0.82, 1.03) | 0.141 |
| Specialized or professional training college | 1.01 (0.89, 1.15) | 0.887 |
| Technical college or junior college | 1.01 (0.79, 1.29) | 0.946 |
| University or more | Reference |  |
| Others | 0.43 (0.05, 3.90) | 0.449 |
| Employment status of wife |  |  |
| Regular worker | Reference |  |
| Non–regular worker | 1.09 (0.98, 1.23) | 0.120 |
| Self–employed worker | 0.99 (0.79, 1.23) | 0.926 |
| Unemployed person | 1.14 (1.01, 1.29) | 0.037 |
| Employment status of husband |  |  |
| Regular worker | Reference |  |
| Non–regular worker | 0.73 (0.49, 1.08) | 0.115 |
| Self–employed worker | 1.04 (0.90, 1.20) | 0.608 |
| Unemployed person | 0.89 (0.48, 1.64) | 0.703 |
| Household income |  |  |
| Quantile 1(Lowest) | 1.05 (0.90, 1.23) | 0.503 |
| Quantile 2 | 1.07 (0.92, 1.24) | 0.372 |
| Quantile 3 | 1.11 (0.96, 1.28) | 0.172 |
| Quantile 4 (Highest) | Reference |  |
| PR, prevalence ratio; CI, confidence interval |  |  |
| ^a^ PR represents the prevalence ratio of modern contraceptive use for each category relative to the reference category. | | |
